# Metatranscriptomic Analysis of Larval Guts from Field-Collected and Laboratory-Reared *Spodoptera frugiperda* from the South American Subtropical Region

**DOI:** 10.1128/genomeA.00777-15

**Published:** 2015-07-16

**Authors:** Christina B. McCarthy, Natalia A. Cabrera, Eduardo G. Virla

**Affiliations:** a Centro Regional de Estudios Genómicos, Facultad de Ciencias Exactas, Universidad Nacional de La Plata, La Plata, Buenos Aires, Argentina; b Departamento de Informática y Tecnología, Escuela de Ciencias Agrarias, Naturales y Ambientales, Universidad Nacional del Noroeste de la Provincia de Buenos Aires, Pergamino, Buenos Aires, Argentina; c PROIMI-Biotecnologia, División Control Biológico, San Miguel de Tucumán, Tucumán, Argentina; d Instituto de Entomología, Fundación Miguel Lillo, San Miguel de Tucumán, Tucumán, Argentina

## Abstract

This is the first study to report a high-throughput approach integrating gene expression data from *Spodoptera frugiperda* guts and their associated metatranscriptomes. Our datasets provide information on the potential effects of environmental conditions on the expression profile of *S. frugiperda* larval guts, their associated metatranscriptome, and putative interactions between them.

## GENOME ANNOUNCEMENT

*Spodoptera frugiperda* is a noctuid moth that devastates various crops including corn, rice, and cotton and is found in most of the American continent (1). Even though transgenic crops that produce insecticidal proteins from *Bacillus thuringiensis* are currently the most successful biotechnological pest management application (2), *S. frugiperda* has developed field-evolved resistance to Bt maize (3). In this sense, gut microbiota play important roles in the growth and development of insects, and metatranscriptomic analyses of insect hosts represent an invaluable tool which could lead to new targets for pest control (4, 5). Nevertheless, the assessment of metabolically active components in the gut microbiota (6) is surprisingly scarce. Furthermore, comprehensive analyses combining host gene expression data with information from associated microbiota (7), are even more rare. The purpose of this study was to integrate gene expression data from *S. frugiperda* guts and their associated metatranscriptomes, under natural and controlled conditions. For this, four *S. frugiperda* samples from the province of Tucumán (Argentina; subtropical region) were analyzed. Specimens were obtained from different environments, altitudes and food sources, namely, (1) a transgenic maize (*Zea mays*) field at 495 meters above sea level (MASL) where insecticides and fertilizers were applied (named Sf_MM; 26°49′50″S, 65°16′59.4″W), (2) *Sorghum halepense* at 495 MASL (Sf_MS; 26°49′50″S, 65°16′59.4″W), (3) a maize field at 2,283 MASL where no insecticides or fertilizers were used (Sf_TV; 26°55′40.75″S, 65°45′19.90″W), and (4) a colony established from larvae originally collected from the same transgenic maize field as Sf_MM, reared for 9 generations under controlled conditions on an artificial diet adapted from reference (8), without the addition of antibiotics (Sf_LR). For all samples, total RNA extracted from fifth instar larval guts (two digestive tracts per sample), was submitted to a one-step reverse transcription and PCR sequence-independent amplification procedure, modified from reference (9), as described previously (7, 10). High-throughput pyrosequencing of the samples was performed using a Roche GS FLX (Macrogen, Inc., Republic of Korea), yielding ~1 Gb of metatranscriptomic reads with lengths of 50 to 1,600 base nucleotides (nt) (652 nt average).

Raw sequence reads were trimmed to remove nucleotides derived from the amplification primers using a custom application (7, 10). The nonredundant protein sequence NCBI database (DB:nr) was downloaded locally. Rapsearch2 (11) was used for the protein homology search against DB:nr, using the trimmed singlet reads, and the taxonomic and functional content of the datasets was analyzed with MEGAN (12, 13). An average of ~97% of the transcripts showed homology to eukaryota, as expected (less than 1% corresponded to nonlepidopteran transcripts, including fungi, kinetoplastida, and viridiplantae, among others), 0.28% to 0.07% to bacteria, 0.003% to 0.001% to archaea, and 0.03% to 0% to viruses. The highest number of bacterial transcripts was found in Sf_MS, whereas archaeal and viral transcripts were more abundant in Sf_LR. Statistical analysis (Fisher’s exact test [14], *P* < 0.05) mostly indicated significant differences between samples.

This is the first study to report *S. frugiperda* metatranscriptomic datasets, which provide information on the potential effects of food source, insecticides, fertilizers, and environmental conditions on the expression profile of *S. frugiperda* larval guts, their associated metatranscriptome, and putative interactions between them.

### Nucleotide sequence accession numbers.

Nucleotide sequences were submitted to the NCBI Sequence Read Archive (SRA) under accession numbers SRX1000994, SRX1000995, and SRX1000996 (field-collected *S. frugiperda* larval guts) and SRX684522 (laboratory-reared *S. frugiperda* larval guts).
